# Sexual Networks and HIV Risk among Black Men Who Have Sex with Men in 6 U.S. Cities

**DOI:** 10.1371/journal.pone.0134085

**Published:** 2015-08-04

**Authors:** Hong-Van Tieu, Ting-Yuan Liu, Sophia Hussen, Matthew Connor, Lei Wang, Susan Buchbinder, Leo Wilton, Pamina Gorbach, Kenneth Mayer, Sam Griffith, Corey Kelly, Vanessa Elharrar, Gregory Phillips, Vanessa Cummings, Beryl Koblin, Carl Latkin, HPTN 061

**Affiliations:** 1 Laboratory of Infectious Disease Prevention, Lindsley F. Kimball Research Institute, New York Blood Center, New York, NY, United States of America; 2 Division of Infectious Diseases, Department of Medicine, Columbia University Medical Center, New York, NY, United States of America; 3 Vaccine and Infectious Disease Division, Fred Hutchinson Cancer Research Center, Seattle, WA, United States of America; 4 Division of Infectious Diseases, Emory School of Medicine, Atlanta, GA, United States of America; 5 Bridge HIV, San Francisco Department of Public Health, San Francisco, CA, United States of America; 6 Department of Human Development, State University of New York at Binghamton, Binghamton, NY, United States of America; 7 Faculty of Humanities, University of Johannesburg, Johannesburg, South Africa; 8 Department of Epidemiology, School of Public Health, Division of InfectiousDiseases, David Geffen School of Medicine, University of California, Los Angeles, Los Angeles, CA, United States of America; 9 Fenway Community Health Center, Boston, MA, United States of America; 10 FHI 360, Research Triangle Park, NC, United States of America; 11 Division of AIDS, National Institute of Allergy and Infectious Diseases, National Institutes of Health, Bethesda, MD, United States of America; 12 The George Washington University School of Public Health and Health Services, Department of Epidemiology and Biostatistics, Washington, DC, United States of America; 13 Johns Hopkins University School of Medicine, Baltimore, MD, United States of America; 14 Johns Hopkins Bloomberg School of Public Health, Baltimore, MD, United States of America; Centers for Disease Control and Prevention, UNITED STATES

## Abstract

**Background:**

Sexual networks may place U.S. Black men who have sex with men (MSM) at increased HIV risk.

**Methods:**

Self-reported egocentric sexual network data from the prior six months were collected from 1,349 community-recruited Black MSM in HPTN 061, a multi-component HIV prevention intervention feasibility study. Sexual network composition, size, and density (extent to which members are having sex with one another) were compared by self-reported HIV serostatus and age of the men. GEE models assessed network and other factors associated with having a Black sex partner, having a partner with at least two age category difference (age difference between participant and partner of at least two age group categories), and having serodiscordant/serostatus unknown unprotected anal/vaginal intercourse (SDUI) in the last six months.

**Results:**

Over half had exclusively Black partners in the last six months, 46% had a partner of at least two age category difference, 87% had ≤5 partners. Nearly 90% had sex partners who were also part of their social networks. Among HIV-negative men, not having anonymous/exchange/ trade partners and lower density were associated with having a Black partner; larger sexual network size and having non-primary partners were associated with having a partner with at least two age category difference; and having anonymous/exchange/ trade partners was associated with SDUI. Among HIV-positive men, not having non-primary partners was associated with having a Black partner; no sexual network characteristics were associated with having a partner with at least two age category difference and SDUI.

**Conclusions:**

Black MSM sexual networks were relatively small and often overlapped with the social networks. Sexual risk was associated with having non-primary partners and larger network size. Network interventions that engage the social networks of Black MSM, such as interventions utilizing peer influence, should be developed to address stable partnerships, number of partners, and serostatus disclosure.

## Introduction

Black men who have sex with men (MSM) are disproportionately affected by the United States (US) HIV epidemic,[1] despite having fewer sex partners and higher rates of condom use than their White counterparts.[2–5] In light of this seeming paradox, several hypotheses have been advanced to attempt to explain this persistent disparity.[3, 6, 7] One explanation is that differences in sexual network structure and composition place Black MSM at higher risk of HIV acquisition.[3, 7, 8]

Social and sexual networks may influence HIV risk.[9–14] HIV prevalence in a sexual network, and the position of an individual within that network, may have as much effect on a person’s risk for HIV as their own sexual behaviors.[15–17] Characteristics of sexual networks at high risk for transmitting HIV may include increased level of connectivity between individuals (extent to which people are connected, i.e. are having sex with one another), sex partner concurrency (in which sex with one partner takes place between two sex intercourse acts with another partner[18, 19]), and geographical insularity (i.e., proximity based on geography).[20] Additionally, factors such as assortative and disassortative mixing (the extent to which partners are similar to or different from one another based on characteristics such as race/ethnicity and age) have implications for HIV acquisition and transmission.[21] Several studies have shown that Black MSM were more likely to report same-race sex partnerships when compared with MSM of other races and ethnicities.[22–25] Disassortativity by age (having a partner who is older or younger than oneself) has been shown to increase risk by bridging younger and older networks with different HIV prevalence.[26] Some,[22–25, 27] but not all,[28–30] studies have also noted that, compared with non-Black men, Black MSM were more likely to have older sex partners, and that having older partners among Black MSM was associated with HIV risk and unrecognized HIV infection.[25, 31, 32] Limited studies using an egocentric (in which information about sex partners is obtained indirectly from participants)[33] or sociometric (in which participants and all their partners are directly interviewed)[34] approach to social and sexual network analysis have been published among Black MSM.[27, 35–37]

Behaviors, such as unprotected anal sex and partner selection patterns within networks, have been examined in other studies utilizing dyadic or network-level approaches as factors that may heighten HIV risk for Black MSM. One study found that although rates of unprotected anal intercourse (UAI) were similar between Black and White MSM, Black men were more likely to have unprotected sex with a partner of unknown or discordant HIV serostatus.[38] This finding is consistent with other research showing that Black men are less likely to know the HIV status of their partners[39] and less likely to practice serosorting (choosing sex partners with the same HIV status) or seropositioning (HIV negative partner in a discordant relationship taking the anal insertive role, the lower risk position for HIV acquisition among MSM) as HIV risk reduction techniques.[40] However, given a lack of difference in rates of seroadaptive behaviors across race/ethnicity among MSM in another study,[41] more research is needed before making any final conclusions.

This current body of research on seroadaptation strategies among MSM has generated pertinent hypotheses about the ways in which sexual network composition and structure may increase HIV acquisition and transmission risk among Black MSM. Of note, much of the research has been limited to a single geographic context, relatively small numbers of Black MSM within a larger population, and a small number of egocentric or sociometric network studies on sex networks of Black MSM. The aims of this study were to describe the characteristics of sexual networks of Black MSM in six US cities who were enrolled in the HIV Prevention Trials Network (HPTN) 061 study and evaluate network, sociodemographic, and risk behavior factors associated with assortative mixing by race/ethnicity (having sex partners of same race/ethnicity, i.e., Black partners), disassortative mixing by age (having sex partners different in age from oneself), and serodiscordant/serostatus unknown unprotected anal intercourse (SDUI).

## Materials and Methods

The institutional review boards at all participating institutions (i.e., New York Blood Center, San Francisco Department of Public Health, Fenway Community Health Center, Harlem Prevention Center, University of California Los Angeles, Emory University, and George Washington University) approved the study. Participants provided written informed consent for the study.

The HPTN 061 study has been described previously.[42, 43] Briefly, HPTN 061 tested the feasibility and acceptability of a multi-component intervention to prevent HIV infection for Black MSM in Los Angeles and San Francisco, CA; Atlanta, GA; Boston, MA; New York, NY; and Washington, DC. Between 2009–2010, Black MSM were recruited directly from the community or as sex partners referred into the study by community-recruited participants. Methods for recruitment of the community-recruited men were developed by and varied at each site, including community outreach, engagement of key informants and local community-based groups, and print and online advertising. Because the study had a particular interest in enrolling men who were HIV-positive but unaware of their status and men who were HIV-positive but not in care and reported unprotected sex with uninfected partners or partners of unknown status, enrollment caps were created for specific categories of participants. Overall, the enrollment target for each site was 250 community-recruited participants who agreed to HIV testing with a limit of 200 HIV-negative participants. An enrollment cap of 10 was applied to community-recruited participants with a prior HIV diagnosis who were already in care, or reported only having unprotected anal sex with HIV-positive partners. No more than 83 participants per site who refused HIV testing could be enrolled.

Men were eligible for study participation if they self-identified as a man or male at birth; self-identified as Black, African American, Caribbean Black, or multi-ethnic Black; at least 18 years old; and reported at least one episode of UAI with a man in the past six months. At the enrollment visit, staff confirmed eligibility and obtained written informed consent. After providing demographic information to an interviewer, participants completed an audio computer-assisted self-interview (ACASI) behavioral assessment. A social and sexual network questionnaire (SSN) was then completed with an interviewer. All participants received HIV risk-reduction counseling and testing using rapid HIV tests as previously described.[42] Participants testing HIV-positive were referred for medical and social services. The participants were reimbursed with a cash stipend with or without a transportation reimbursement, which varied by site.

### Measures

An interviewer collected basic demographic information, including age (as a continuous variable), self-identified gender, sexual orientation, self-identification as Latino/Hispanic, education, and marital status.

Data on history of incarceration, alcohol and drug use, and self-reported HIV serostatus were collected on ACASI. The Center for Epidemiologic Studies Depression Scale (CES-D) was used to measure depression.[44] A participant with a score of ≥16 was considered to have clinically significant depressive symptoms.

As HIV status knowledge may influence network configuration, the analyses focused on self-reported HIV serostatus of the participant obtained from the ACASI questionnaire at the baseline study visit prior to HIV testing.

### Network questionnaire

For the social network inventory, each participant was asked to name up to five persons whom he could rely on for functional support using four domains.[45, 46] Relationship and sociodemographic questions were asked about each social network member, including whether the social network member was also a sex partner.

For the sexual network inventory, participants were asked about their partners with whom they had anal or vaginal sex in the last six months using a name generator, up to 10 sex partners. If they had more than 10 partners, they were asked to approximate how many additional sex partners. The following questions were asked about each named sex partner: (1) sociodemographics, including age (as categorical variables ≤17 years, 18–20, 21–25, 25–30, 30–40, 40–50, 50–60, and ≥60), gender, race/ethnicity, (2) perceived HIV status of partner, (3) HIV disclosure to partner among HIV-positive participants, (4) sex partner type, and (5) frequency of anal (receptive or insertive) or vaginal sex and condom use with the partner in the last six months. A network density matrix was completed that captured information about any sexual relationships among each partner named. Sex partner type was categorized as follows: (1) primary partner, (2) steady, non-primary partner, (3) casual partner, (4) exchange or trade partner, and (5) anonymous partner.

### Network-derived variables

The racial/ethnic composition of the sex partners of the participants was classified as exclusively Black, exclusively non-Black, or both Black and non-Black. Since age of sex partners was asked as a categorical variable, age difference between the participants and sex partners was categorized as having no partner with at least two age group category difference vs. having a partner with at least two age group category difference. Sexual network size was calculated by summing the total number of people in the sexual network in the last six months, including the participant, social partners who were also sex partners, enumerated sex partners, and number of additional sex partners beyond the named partners. Sexual network density was calculated,[47] and refers to the extent to which members of the sexual network, excluding the participant, are interconnected (i.e., having sex with one another). Density values could vary from 0% (no partner is sexually linked to any other member of participant’s sexual network) to 100% (all partners are sexually linked to one another).[47, 48] Presence or absence of any overlap between social and sexual networks was determined based on whether the participants specified any members of the social networks who were also sex partners. Assortative mixing patterns were examined by age categories using the Newman assortativity coefficient derived from the mixing matrix.[49] Based on previous research, a mixing coefficient value of > 0.35 was considered assortative, 0.26–0.34 moderately assortative, 0.15–0.25 minimally assortative, and <0.15 discordant.[50, 51]

Number of female sex partners in the participants’ sexual networks was categorized as either none or at least one. SDUI referred to having unprotected anal and/or vaginal intercourse with a male or female sex partner in the last six months with HIV serodiscordance or serostatus unknown, and was dichotomized as any or no SDUI. Based on the participant's belief about their partner’s HIV serostatus, a sexual event was considered serodiscordant/serostatus unknown if the partner’s HIV status was unknown or different from self-reported HIV status of the participant on the ACASI questionnaire at the baseline visit.[28]

### Statistical Methods

Only community-recruited participants were included in this analysis, with exclusion of referred sex partners because of the concern for correlation of sexual network variables of referred participants. Participant and sex network characteristics were compared by self-reported HIV serostatus and age groups (18–30 years vs. >30 years) using Chi-Square test or Fisher’s exact test. For the partner-level comparison, characteristics of the sex partners were compared by self-reported HIV serostatus of the participants using Chi-Square test. Associations between participant characteristics (e.g., age, gender), sex partner characteristics (e.g., age, gender, partner type), and sex network characteristics (e.g., network size, density) were assessed for three outcomes of interest, stratified by self-reported HIV serostatus of the participant: having a Black sex partner, having a partner with at least two age category difference, and having SDUI in the last six months. These three outcomes were partner-level variables, with each partner included as a separate observation. To account for correlations among multiple partners of the same participant in the models, multivariate Generalized Estimating Equation (GEE) methods were used. Six GEE models were constructed, with three models for each self-reported HIV status of the participant. The GEE models controlled for study city, since site-specific differences may be reflective of different recruitment strategies used by the sites, rather than of overall differences between cities. Adjusted odds ratio (AOR) was calculated, as well as 95% confidence intervals. A p-value of <0.05 was considered statistically significant. Analyses were conducted using SAS version 9.2.

## Results

### Baseline Participant, Partner, and Sexual Network Characteristics

A total of 1,349 community-recruited men enrolled in the study. Overall, 91% self-reported being HIV-negative and 9% HIV-positive (Table 1). Of the 123 men who self-reported being HIV-positive and were tested at the baseline visit using on-site rapid HIV tests, 92% tested HIV-positive. Of the 1,066 men who self-reported being HIV-negative and were tested at the baseline visit, 97% tested HIV-negative and 3% tested HIV-positive. Concordance between self-reported HIV status and HIV test results was high (kappa coefficient 0.81). Fifty-two percent reported SDUI with a male or female partner in the last six months; 55% reported having exclusively Black sex partners and 46% reported having a partner with at least a two age category difference between the participant and partner. Most of the men reported having a sexual network size of fewer than six partners in the last six months; 88% reported a sexual network density of 0%; and 87% reported having sex partners who were also a part of their social networks.

**Table 1:** Sociodemographics, Risk Behaviors, and Sexual Network Characteristics of Community-Recruited Black MSM Stratified by Self-Reported HIV Serostatus and Age, Participant- and Network-Level Data (N = 1,349 Participants).

Characteristic, n (%)^a^		Self-Reported HIV-Positive Participants				Self-Reported HIV-Negative Participants Total				HIV Status P-value^b^
			Age Stratification of Participants				Age Stratification of Participants			
	Overall Total	Self-Reported HIV-Positive Participants Total	18–30 years	31+ years	P-value	Self-Reported HIV-Negative Participants Total	18–30 years	31+ years	P-value	
Number of Participants	1349 (100)	123 (9)	21 (17)	102 (83)		1226 (91)	456 (37)	770 (63)		NA
**Participant Level**										
Age (years)										**<0.01**
18–20	98 (7)	1 (1)	NA	NA	NA	97 (8)	NA	NA	NA	
21–30	379 (28)	20 (16)				359 (29)				
31–40	241 (18)	32 (26)				209 (17)				
>40	631 (47)	70 (57)				561 (46)				
Gender					0.67				0.32	0.23
Male	1324 (98)	119 (97)	20 (95)	99 (97)		1205 (98)	446 (98)	759 (99)		
Transgender	25 (2)	4 (3)	1 (5)	3 (3)		21 (2)	10 (2)	11 (1)		
Sexual orientation					0.33				**<0.01**	**<0.01**
Homosexual/gay	397 (30)	49 (40)	11 (52)	38 (38)		348 (29)	162 (36)	186 (25)		
Bisexual	374 (28)	20 (17)	4 (19)	16 (16)		354 (29)	86 (19)	268 (35)		
Other	558 (42)	52 (43)	6 (29)	46 (46)		506 (42)	201 (45)	305 (40)		
Marital status					0.53				0.08	**0.01**
Married or living with partner	102 (8)	17 (14)	2 (10)	15 (15)		85 (7)	24 (5)	61 (8)		
Single/divorced/widowed/not living with partner	1246 (92)	106 (86)	19 (90)	87 (85)		1140 (93)	432 (95)	708 (92)		
Latino/Hispanic					0.05				**<0.01**	0.21
Yes	113 (8)	14 (11)	5 (24)	9 (9)		99 (8)	52 (11)	47 (6)		
No	1236 (92)	109 (89)	16 (76)	93 (91)		1127 (92)	404 (89)	723 (94)		
Education					0.25				**0.01**	0.07
Less than college degree	706 (52)	55 (45)	7 (33)	48 (47)		651 (53)	221 (49)	430 (56)		
College degree or higher	642 (48	68 (55)	14 (67)	54 (53)		574 (47)	234 (51)	340 (44)		
Any SDUI^c^ with a male or female partner in past 6 months					0.16				**<0.01**	0.78
Yes	697 (52)	65 (53)	14 (67)	51 (50)		632 (52)	196 (43)	436 (57)		
No	652 (48)	58 (47)	7 (33)	51 (50)		594 (48)	260 (57)	334 (43)		
**Network Level**										
Race/ethnicity of sexual partners					0.48				0.31	0.51
Exclusively Black	740 (55)	74 (61)	11 (52)	63 (62)		666 (55)	236 (52)	430 (56)		
Exclusively non-Black	186 (14)	17 (14)	2 (10)	15 (15)		169 (14)	67 (15)	102 (13)		
Both Black and non-Black	416 (31)	31(25)	8 (38)	23 (23)		385 (32)	153 (34)	232 (30)		
≥2 age category difference between participant and sex partners^d^					0.97				**<0.01**	0.68
No partner	725 (54)	64 (52)	11 (52)	53 (52)		661 (54)	276 (61)	385 (50)		
≥1 partner	622 (46)	59 (48)	10 (48)	49 (48)		563 (46)	180 (39)	383 (50)		
Sexual network size					**0.04**				**0.03**	**<0.01**
1	272 (20)	43 (35)	4 (19)	39 (38)		229 (19)	85 (19)	144 (19)		
2	325 (24)	29 (24)	3 (14)	26 (25)		296 (24)	90 (20)	206 (27)		
3–5	572 (42)	38 (31)	9 (43)	29 (28)		534 (44)	209 (46)	325 (42)		
≥6	180 (13)	13 (11)	5 (24)	8 (8)		167 (14)	72 (16)	95 (12)		
Sexual network density (n = 1077)					0.06				0.14	0.54
0%	1183 (88)	105 (85)	15 (71)	90 (88)		1078 (88)	401 (88)	677 (88)		
0< % <50	122 (9)	12 (10)	5 (24)	7 (7)		110 (9)	46 (10)	64 (8)		
50≤ % ≤100	44 (3)	6 (5)	1 (5)	5 (5)		38 (3)	9 (2)	29 (4)		
Any overlap between sexual and social networks					0.41				0.08	0.31
Yes	1180 (87)	104 (85)	19 (90)	85 (83)		1076 (88)	410 (90)	666 (86)		
No	169 (13)	19 (15)	2 (10)	17 (17)		150 (12)	46 (10)	104 (14)		
Number of female partners in sexual networks					0.77				**<0.01**	**<0.01**
None	935 (69)	109 (89)	19 (90)	90 (88)		826 (67)	374 (82)	452 (59)		
≥1	412 (31)	14 (11)	2 (10)	12 (12)		398 (33)	82 (18)	316 (41)		

Numbers may not add up to total due to missing data.

NA: Not applicable.

^a^ P-value <0.05 for all variables when compared by study city, except for self-reported HIV serostatus at enrollment. Site-specific differences may be reflective of different recruitment strategies used by the study sites, rather than of overall differences between cities.

^b^ P-value comparing self-reported HIV-positive men with self-reported HIV-negative men

^c^ SDUI: serodiscordant/serostatus unknown unprotected anal and/or vaginal intercourse

^d^ Age category difference is based on the following age categorical variables of the sexual partners in the social and sexual network inventory: ≤17 years, 18–20, 21–25, 25–30, 30–40, 40–50, 50–60, and ≥60

Compared with self-reported HIV-negative men, self-reported HIV-positive men were more likely to be older, self-identify as homosexual and gay, be married or living with a partner, have fewer sex partners, and have no female sex partners in the last six months. Among HIV-negative men, older men (>30 years) were more likely to report SDUI, have a sex partner of at least 2 age category difference, have smaller sexual network size, and have a female partner in the last six months compared with younger men (18–30 years). Among HIV-positive men, older men were more likely to have a smaller sexual network size compared with younger men.

Over three-quarters of the sex partners of community-recruited participants were male, 18% female, and 4% transgender (Table 2). Sex partners of HIV-positive participants were more likely to be male and less likely to be female than partners of HIV-negative participants. Partners of HIV-positive participants were more likely to be HIV-positive and less likely to be HIV-negative than partners of HIV-negative participants. HIV-positive men reported not disclosing their HIV status to 34% of their partners.

**Table 2:** Sex Partner Characteristics, Condom Use, and HIV Serostatus Disclosure of Community-Recruited Black MSM, Partner-Level Data (N = 4,449 Partners).

Characteristic, n (%)^a^	Total	Self-Reported HIV-Positive Participant	Self-Reported HIV-Negative Participant	P-value
**Partner Level**				
Gender of sex partners				**<0.01**
Male	3464 (78)	308 (92)	3156 (77)	
Female	800 (18)	18 (5)	782 (19)	
Transgender	172 (4)	7 (2)	165 (4)	
Partner type				**<0.01**
Primary partner	707 (16)	80 (24)	627 (15)	
Steady, non-primary partner/casual partner	3003 (68)	210 (63)	2793 (68)	
Exchange or trade partner/anonymous partner	716 (16)	42 (13)	674 (16)	
HIV serostatus of sex partners				**<0.01**
Among all participants (N = 4446 partners):	2284 (51)	67 (20)	2217 (54)	
HIV-negative	311 (7)	142 (43)	169 (4)	
HIV-positive	1841 (41)	124 (37)	1717 (42)	
HIV serostatus of sex partners				**<0.01**
Among participants age 18–30 years (N = 1659 partners):	1023 (62)	17 (23)	1006 (63)	
HIV-negative	66 (4)	22 (30)	44 (3)	
HIV-positive	570 (34)	35 (47)	535 (34)	
HIV serostatus of sex partners				**<0.01**
Among participants age 31+ years (N = 2777 partners):	1261 (45)	50 (19)	1211 (48)	
HIV-negative	245 (9)	120 (46)	125 (5)	
HIV-positive	1271 (46)	89 (34)	1182 (47)	
Frequency of condom use with sex in past 6 months (N = 4361 partners)				**0.01**
Never	1899 (44)	171 (52)	1728 (43)	
Sometimes	740 (17)	52 (16)	688 (17)	
Most of the time	398 (9)	18 (5)	380 (9)	
Always	1324 (30)	88 (27)	1236 (31)	
Disclosure of HIV status to sex partners by self-reported HIV-positive participants (N = 309 partners)	-		-	-
No		106 (34)		
Yes		191 (62)		
Don’t know/refused to answer		12 (4)		

Numbers may not add up to column total due to missing data.

^a^ P-value <0.05 for all variables compared by study city. Site-specific differences may be reflective of different recruitment strategies used by the study sites, rather than of overall differences between cities.

The assortative mixing coefficient by age was 0.20 (considered minimally assortative).[50, 51]

### Multivariate GEE Logistic Regression Models

Multivariate models for each outcome stratified by self-reported HIV status are presented in Table 3. The odds of having a Black partner among HIV-positive men were higher with not identifying as Latino/Hispanic, while lower for having a non-primary partner. Among HIV-negative men, the odds of having a Black partner were higher with age ≤20 years vs. >40 years, self-identifying as bisexual vs. homosexual/gay, not identifying as Latino/Hispanic, and having less than college degree. The odds were lower for men aged between 21–40 years vs. >40 years, those having an anonymous/exchange or trade partner vs. primary partner, and those having a sexual network density of 50–100% vs. 0%.

**Table 3:** Participant and Sex Network Characteristics Associated with Having a Black Sex Partner, Having a Partner with at Least 2 Age Category Difference, and Having SDUI in the Last 6 Months among Community-Recruited Participants Stratified by Self-Reported HIV Serostatus of Participant, Multivariate GEE Models (N = 4,449 Partners).

	Having a Black Sex Partner in the Last 6 Months		Having a Partner with at Least 2 Age Category Difference in the Last 6 Months		Having SDUI in the Last 6 Months	
	Self-Reported HIV-Positive	Self-Reported HIV-Negative	Self-Reported HIV-Positive	Self-Reported HIV-Negative	Self-Reported HIV-Positive	Self-Reported HIV-Negative
	Multivariate	Multivariate	Multivariate	Multivariate	Multivariate	Multivariate
Participant-Level Characteristics	AOR (95% CI)	AOR (95% CI)	AOR (95% CI)	AOR (95% CI)	AOR (95% CI)	AOR (95% CI)
Age of participant (years)			Not tested	Not tested		
≤20	NA	**1.71 (1.12, 2.60)**			NA	0.67 (0.43, 1.05)
21–30	0.84 (0.28, 2.54)	**0.65 (0.50, 0.85)**			0.43 (0.14, 1.30)	**0.46 (0.35, 0.62)**
31–40	0.77 (0.31, 1.92)	**0.63 (0.47, 0.84)**			0.57 (0.23, 1.44)	0.96 (0.71, 1.29)
>40	Ref	Ref			Ref	Ref
Gender of participant						
Male	Ref	Ref	Ref	Ref	Ref	Ref
Transgender	1.12 (0.20, 6.33)	1.63 (0.60, 4.30)	0.96 (0.20, 4.75)	0.98 (0.45, 2.14)	0.73 (0.11, 4.86)	1.35 (0.61, 2.98)
Sexual orientation						
Homosexual/gay	Ref	Ref	Ref	Ref	Ref	Ref
Bisexual	0.42 (0.13, 1.35)	**1.50 (1.13, 2.00)**	1.35 (0.52, 3.52)	0.86 (0.65, 1.13)	0.89 (0.29, 2.73)	0.99 (0.73, 1.34)
Other	0.59 (0.24, 1.47)	1.01 (0.77, 1.32)	1.28 (0.59, 2.80)	1.00 (0.78, 1.28)	1.10 (0.47, 2.59)	1.01 (0.78, 1.31)
Latino/Hispanic			Not tested	Not tested	Not tested	Not tested
Yes	Ref	Ref				
No	**3.58 (1.33, 9.59)**	**1.76 (1.27, 2.43)**				
Marital status						
Married or living with partner	Ref	Ref	Ref	Ref	Ref	Ref
Not living with partner or single, divorced, or widowed	1.38 (0.41, 4.64)	1.42 (0.94, 2.15)	0.74 (0.25, 2.18)	0.95 (0.63, 1.43)	2.10 (0.78, 5.68)	1.44 (0.92, 2.26)
Education						
Less than college degree	1.63 (0.69, 3.85)	**1.26 (1.02, 1.55)**	1.18 (0.63, 2.23)	1.00 (0.82, 1.23)	1.06 (0.50, 2.27)	**1.27 (1.01, 1.60)**
College degree or higher	Ref	Ref	Ref	Ref	Ref	Ref
Depression	Not tested	Not tested	Not tested	Not tested		
Yes					**2.72 (1.19, 6.21)**	1.01 (0.81, 1.26)
No					Ref	Ref
Any drug or alcohol use before unprotected sex in the last 6 months	Not tested	Not tested	Not tested	Not tested		
Yes					1.09 (0.48, 2.49)	**1.28 (1.01, 1.63)**
No					Ref	Ref
**Partner Characteristics**						
Partner age (years)			Not tested	Not tested		
≤20	0.79 (0.16, 3.79)	0.89 (0.62, 1.29)			2.07 (0.52, 8.30)	0.83 (0.61, 1.12)
21–30	0.87 (0.38, 1.99)	0.78 (0.63, 0.97)			**2.49 (1.24, 5.00)**	1.04 (0.88, 1.25)
31–40	0.88 (0.45, 1.73)	0.87 (0.71, 1.07)			**2.30 (1.27, 4.14)**	1.04 (0.88, 1.22)
>40	Ref	Ref			Ref	Ref
Gender of partner						
Male	Ref	Ref	Ref	Ref	Ref	
Female	0.78 (0.29, 2.15)	0.83 (0.67, 1.02)	0.48 (0.14, 1.72)	0.90 (0.72, 1.12)	1.53 (0.44, 5.27)	Ref
Transgender	0.55 (0.04, 7.84)	0.75 (0.50, 1.13)	**12.39 (1.40, 109.9)**	1.41 (0.98, 2.04)	0.61 (0.08, 4.40)	0.86 (0.73, 1.02)
Race of partner	Not applicable	Not applicable				
Non-Black			1.52 (0.83, 2.78)	**1.32 (1.12, 1.54)**	1.41 (0.74, 2.70)	0.90 (0.76, 1.06)
Black			Ref	Ref	Ref	Ref
Type of sexual partner						
Primary	Ref	Ref	Ref	Ref	Ref	Ref
Casual/steady	**0.36 (0.18, 0.76)**	0.85 (0.70, 1.03)	2.03 (0.92, 4.47)	**1.41 (1.14, 1.75)**	0.81 (0.42, 1.55)	1.10 (0.91, 1.34)
Anonymous/exchange or trade	**0.27 (0.09, 0.83)**	**0.70 (0.53, 0.91)**	1.75 (0.55, 5.52)	**1.47 (1.08, 2.00)**	1.47 (0.55, 3.89)	**1.31 (1.01, 1.71)**
**Sexual Network Characteristics**						
Sexual network size						
1 partner	Ref	Ref	Ref	Ref	Ref	Ref
2	0.56 (0.15, 2.02)	0.97 (0.65, 1.45)	0.69 (0.24, 2.03)	1.21 (0.80, 1.84)	1.61 (0.58, 4.47)	1.20 (0.81, 1.78)
3–5	0.54 (0.16, 1.75)	0.99 (0.69, 1.43)	0.82 (0.32, 2.07)	1.33 (0.90, 1.95)	1.05 (0.36, 3.12)	1.25 (0.87, 1.79)
≥6	2.25 (0.55, 9.19)	0.76 (0.50, 1.15)	0.71 (0.23, 2.13)	**1.71 (1.13, 2.61)**	1.22 (0.27, 5.41)	0.91 (0.59, 1.40)
Sexual network density						
0%	Ref	Ref	Ref	Ref	Ref	Ref
0< % <50	1.11 (0.36, 3.44)	0.85 (0.62, 1.17)	1.43 (0.61, 3.32)	1.24 (0.91, 1.68)	1.19 (0.33, 4.26)	0.89 (0.61, 1.29)
50≤ % ≤100	1.78 (0.20, 15.67)	**0.60 (0.36, 0.99)**	0.39 (0.10, 1.47)	1.51 (0.82, 2.80)	0.27 (0.07, 1.07)	1.24 (0.63, 2.41)
Any overlap of social and sexual networks						
No	0.93 (0.49, 1.75)	0.96 (0.81, 1.14)	0.93 (0.51, 1.69)	1.00 (0.85, 1.17)	0.78 (0.44, 1.35)	1.11 (0.97, 1.27)
Yes	Ref	Ref	Ref	Ref	Ref	Ref

GEE: Generalized estimating equation, SDUI: serodiscordant/serostatus unknown unprotected anal and/or vaginal intercourse, AOR: Adjusted odds ratio, 95% CI: 95% Confidence Interval, Ref: Reference, NA: Not applicable as there were no observations detected.

The odds of having a partner with at least two age category difference among HIV-positive men were higher with having a transgender partner vs. male partner. The odds of having a partner with at least two age category difference among HIV-negative men were higher with having a non-Black partner, having a non-primary partner vs. primary partner, and having a sexual network size ≥6 vs. 1 partner.

The odds of having SDUI in the last six months among HIV-positive participants were higher with having depression and having a partner between 21–40 years of age vs. >40 years. Among HIV-negative men, the odds of SDUI were lower with younger age, while higher for those having less than a college degree, any drug/alcohol use before unprotected sex in the last six months, and having an anonymous/exchange or trade partner vs. primary partner.

## Discussion

In this large cohort of Black MSM in the US involving detailed egocentric network data collection, the sexual networks of Black MSM tended to be relatively small compared with other cohorts consisting of more racially diverse MSM.[52, 53] However, estimates of network size by an egocentric network approach, used in our study as well as other studies, may underestimate true network size. Even though many of the men’s sexual networks were small and network size was not associated with SDUI, men with larger network size have the potential to engage in sexual relationships with known or undiagnosed HIV-positive men within smaller sexual networks, leading to linkage of networks and greater HIV acquisition and transmission risk across networks.

Higher degrees of racial/ethnic assortativity are thought to portend increased HIV risk to Black MSM given the higher HIV prevalence (and higher prevalence of men with undiagnosed HIV infection and men with HIV infection who are not on antiretroviral treatment[54–58]) in those networks. We found that slightly more than half of men reported having exclusively Black sex partners in the last six months, with nearly a third reporting having both Black and non-Black partners. Network studies have shown that Black MSM were more likely to report same-race sex partnerships when compared with MSM of other races and ethnicities.[22–26, 28–30] In our study, we found that having a Black sex partner was not significantly associated with sexual network size and overlap of social and sexual networks for both HIV-positive and HIV-negative men. HIV-negative men who reported a high sex network density were less likely to have a Black partner; no association between sex network density and having a Black partner was observed for HIV-positive men.

Although Black men were more likely to have Black sex partners, we did not find evidence that the sexual networks of the men were dense; this, however, might merely reflect a limitation of the egocentric network design in that the men might not necessarily have accurate knowledge about sexual relationships and encounters between their sex partners. We also did not find race/ethnicity of the partner to be significantly associated with SDUI for both HIV-positive and HIV-negative participants. Therefore, it is likely that other factors, such as HIV prevalence within sex networks of Black MSM, are driving transmission of HIV in this population.

Almost half of the men reported having a partner with at least two age category difference. The assortative mixing coefficient by age was 0.20, suggesting that sexual mixing by age was minimally assortative.[50, 51] Although several studies have shown an association between having older sex partners and HIV risk among young Black MSM,[25, 31, 32] findings from other studies have not supported this association.[5, 7, 39] In our study, having a younger partner (i.e., partner between 21–40 years), as opposed to having an older partner >40 years, was significantly associated with SDUI among HIV-positive men. This association was not seen among HIV-negative men. Our findings differ somewhat from a study which found that having an older partner was associated with having unprotected anal or vaginal sex among Black MSM, and that this association was more robust as the participant’s age decreased.[26] In our study, a large sexual network size of six or more partners was significantly associated with having a partner with at least two age category difference for HIV-negative men only. Having a large number of partners may just increase the opportunity for the men to select partners of different ages, including those with substantial age differences.

A substantial proportion of men reported never using condoms during sex in the last six months. This salient finding emphasizes the importance of more effective messaging and prevention strategies, including large-scale roll out of HIV pre-exposure prophylaxis for HIV-negative men and implementation of ‘treatment as prevention’ paradigm for HIV-positive men. Over half of the men reported having SDUI with a male or female partner, with no difference between self-reported HIV serostatus of the men. This finding highlights the importance of reaching out to both HIV-positive and HIV-negative men to encourage HIV serostatus discussion and accurate disclosure with partners. The SDUI prevalence is higher than that reported in previous studies among Black MSM.[28, 30] In a New York City HIV behavioral intervention study, 27% of Black MSM reported having SDUI during last sex.[28] The difference in SDUI prevalence might be explained by the different timeframe in which SDUI is defined (last six months in our study vs. at last sex in the two studies) and the eligibility criterion in our study of having reported UAI in the six months prior to study enrollment. In another study, 23% of Black MSM reported having serodiscordant unprotected anal sex with a nonmain male partner and 9% with a main male partner in the last 12 months.[30] This is consistent with our study finding that HIV-negative men with an anonymous, exchange, or trade partner were more likely to have SDUI compared with men with a primary partner. We found that depression was associated with having SDUI among HIV-positive men only. Prior research focused on syndemics among MSM, in which psychosocial issues such as depression and substance use interact to increase men’s risk for HIV acquisition and transmission.[59–62] Our finding reinforces the need for accessible mental health services for HIV-positive Black MSM to reduce transmission risk behaviors.

We found that among both self-reported HIV-negative and HIV-positive men, SDUI was not associated with absence of overlap of social and sexual networks. However, because a large proportion of the men reported overlap of social and sexual networks, utilizing social networks to exert normative pressures to reduce HIV risk behaviors (e.g., consistent condom use or disclosure of HIV serostatus) and disseminate HIV prevention messages should be explored.[51] Our finding that HIV-negative men who did not have an anonymous, exchange, or trade partner were less likely to have SDUI underscores the need for structural interventions that support primary partnerships within Black communities.

Our finding that younger men were less likely to have SDUI may be a positive sign of change in risk behaviors in the younger generations of Black MSM. The negative association between age and SDUI in our study, however, is surprising given the higher HIV infection rates reported nationally among young Black MSM compared with their older counterparts as well as higher HIV incidence rates reported in our longitudinal study among young Black MSM compared with older men.[42] This could in part be explained by assumptions about partner HIV serostatus; we found that younger men were more likely to report having HIV-negative partners and less likely to report having unknown status partners compared with older men. Our result that HIV-positive men who had a female partner were not more likely to report SDUI compared with men who had a male partner is encouraging for preventing new HIV infections in women who are in concurrent relationships with their bisexual Black male partners; this finding differs from that reported in another study that MSM were 4.59 times more likely to have unprotected anal or vaginal sex with female partners than with male partners, though this finding included both Black MSM and MSM of other races/ethnicities.[26]

It is quite concerning that we found a large proportion of partners whose HIV status is unknown to the participants in the study, with 41% of partners of all participants having unknown status. Disclosure of HIV status was 62% among HIV-positive men, with the men not disclosing their HIV to more than a third of their partners. Prior studies have noted comparable rates of HIV serostatus disclosure among Black MSM. In a study among Black MSM in NYC, overall disclosure of HIV serostatus by participants during last sex with a male partner was 67%; 56% of Black MSM reported that their male partners disclosed their HIV serostatus to them during last sex.[28] In another study, 52% of Black MSM reported knowledge of the HIV status of their most recent non-main sex partner, while a third reported knowing the HIV serostatus of their most recent main partner.[30] In a large national study of internet-using MSM, Black men, and in particular Black HIV-positive men, were less likely to discuss their HIV serostatus with partners whom they had unprotected anal sex with than their White counterparts.[63] The relatively low disclosure rates of HIV serostatus by HIV-positive Black men and by partners of both HIV-positive and HIV-negative Black men in our study might help explain the greater HIV transmission and acquisition risk of HIV among Black MSM. The finding also underscores the importance of developing culturally relevant interventions to encourage communication of HIV serostatus to sex partners among Black MSM, including accurate disclosure of HIV serostatus and treatment status among HIV-infected men, and of implementing strategies to reduce HIV stigma in the Black MSM community.

There are limitations to this study. First, the study sample, especially with enrollment caps on specific HIV status categories in the main study design and exclusion of referred participants, might not be representative of all Black MSM in the US. Second, there is the issue of socially desirable responding and misclassification bias. Because the SSN was administered by an interviewer, there is a potential for distortion of self-report of risk behaviors and sex network members. There is the limitation of self-report, especially in regard to HIV serostatus of partners, in this cohort.[64, 65] The number of partners that the participants could name in the SSN was capped at 10 to reduce inaccurate recall and participant fatigue; however, this cap, as well as the few number of name generating questions, could have led to underreporting bias, which might bias true sex network density as well as other network measures. In this study’s egocentric network design, the participants were asked details about their partners, and their partners were not directly interviewed. The participants most likely did not know with certainty about the information about their network members, especially about their anonymous, exchange, and trade sex partners or about actual sexual relations between their named sex partners, and thus were likely imperfect reporters of these factors. In particular, the sexual network density measure and HIV serostatus of partners within networks might have measurement bias (i.e., might underestimate true network density and proportion of HIV-positive partners) because the participants might not have direct knowledge of other sex relations of his partners and true HIV serostatus of partners. In addition, because we were not able to link the network members among the participants, we lacked information whether the networks of the participants overlapped. There is the also the limitation of recall bias, with potentially inaccurate recall of all sex partners during the six-month period. This analysis did not explore geographic or city differences in sexual networks, given that most differences in sexual network characteristics might be due to different recruitment strategies used by the study sites rather than due to any significant cultural differences between Black MSM communities and experiences in the different cities. Lastly, because the ages of the partners were recorded on the SSN in specific age categories, we were not able to accurately determine real age differences between the participants and partners, though this likely would not alter our findings.

## Conclusions

This HPTN 061 investigation is the largest published study on sexual networks of Black MSM in the US and represents an important advancement in the understanding of the influence of sexual networks on HIV risk among Black MSM. Network-based interventions that engage the social networks of Black MSM should be developed to address sexual partnering and HIV transmission risk behaviors to lower HIV incidence rates in this population. Specifically, interventions may utilize peer influence among key members of social networks of Black MSM to encourage frequent HIV testing, use of HIV prevention methods such as condom use and pre-exposure prophylaxis, and linkage to care, antiretroviral treatment initiation and adherence, and retention in HIV care. In addition, counseling of HIV-positive men about reducing HIV transmission risk behaviors, specifically HIV serostatus disclosure, and implementation of strategies to reduce HIV stigma should be emphasized. Community-based programs should be developed to strengthen support and friendship networks among Black MSM and to foster health promotion norms within these networks. Lastly, our findings reinforce the need to develop structural interventions that support maintaining primary partnerships within Black communities.
